# Cost Analysis of a Nucleic Acid Amplification Test in the Diagnosis of Pulmonary Tuberculosis at an Urban Hospital with a High Prevalence of TB/HIV

**DOI:** 10.1371/journal.pone.0100649

**Published:** 2014-07-11

**Authors:** Max W. Adelman, Ekaterina Kurbatova, Yun F. Wang, Michael K. Leonard, Nancy White, Deborah A. McFarland, Henry M. Blumberg

**Affiliations:** 1 Emory University School of Medicine, Atlanta, Georgia, United States of America; 2 Division of Infectious Diseases, Department of Medicine, Emory University School of Medicine, Atlanta, Georgia, United States of America; 3 Hubert Department of Global Health, Rollins School of Public Health, Emory University, Atlanta, Georgia, United States of America; 4 Clinical Microbiology Laboratory, Grady Memorial Hospital, Atlanta, Georgia, United States of America; 5 Department of Pathology and Laboratory Medicine, Emory University School of Medicine, Atlanta, Georgia, United States of America; 6 Department of Epidemiology, Grady Memorial Hospital, Atlanta, Georgia, United States of America; 7 Department of Epidemiology, Rollins School of Public Health, Emory University, Atlanta, Georgia, United States of America; San Francisco General Hospital, University of California San Francisco, United States of America

## Abstract

**Introduction:**

The Centers for Disease Control and Prevention has recommended using a nucleic acid amplification test (NAAT) for diagnosing pulmonary tuberculosis (TB) but there is a lack of data on NAAT cost-effectiveness.

**Methods:**

We conducted a prospective cohort study that included all patients with an AFB smear-positive respiratory specimen at Grady Memorial Hospital in Atlanta, GA, USA between January 2002 and June 2008. We determined the sensitivity, specificity, and positive and negative predictive value of a commercially available and FDA-approved NAAT (amplified MTD, Gen-Probe) compared to the gold standard of culture. A cost analysis was performed and included costs related to laboratory tests, hospital charges, anti-TB medications, and contact investigations. Average cost per patient was calculated under two conditions: (1) using a NAAT on all AFB smear-postive respiratory specimens and (2) not using a NAAT. One-way sensitivity analyses were conducted to determine sensitivity of cost difference to reasonable ranges of model inputs.

**Results:**

During a 6 1/2 year study period, there were 1,009 patients with an AFB smear-positive respiratory specimen at our public urban hospital. We found the NAAT to be highly sensitive (99.6%) and specific (99.1%) on AFB smear-positive specimens compared to culture. Overall, the positive predictive value (PPV) of an AFB smear-positive respiratory specimen for culture-confirmed TB was 27%. The PPV of an AFB smear-positive respiratory specimen for culture-confirmed TB was significantly higher for HIV-uninfected persons compared to those who were HIV-seropositive (152/271 [56%] vs. 85/445 [19%]; RR = 2.94, 95% CI 2.36–3.65, p<0.001). The cost savings of using the NAAT was $2,003 per AFB smear-positive case.

**Conclusions:**

Routine use of the NAAT on AFB smear-positive respiratory specimens was highly cost-saving in our setting at a U.S. urban public hospital with a high prevalence of TB and HIV because of the low PPV of an AFB smear for culture-confirmed TB.

## Introduction

The U.S. Centers for Disease Control and Prevention (CDC) has recommended that a nucleic acid amplification test (NAAT) be used routinely for the diagnosis of pulmonary tuberculosis (TB) [1]. However, few studies have assessed the cost-effectiveness of the routine use of a NAAT such as the amplified MTD test (Gen-Probe, San Diego, CA), which is one of only two available FDA-approved, commercial NAATs in the U.S. Several prior studies on NAAT cost-effectiveness concluded that these tests are not cost-effective in high-income countries with low burdens of TB [2]–[4].

Grady Memorial Hospital (GMH) is 1,000-bed urban university-affiliated public teaching hospital located in a high TB incidence area in Atlanta, GA, USA. GMH provides care to a large number of persons living with HIV. The annual number of TB cases at GMH gradually decreased from >150 in the late 1990s [5] to 40–80 in the last 5 years. Up to 40% of patients with TB at GMH are HIV co-infected. Additionally, there is a large burden of disease due to non-tuberculous mycobacteria (NTM) at GMH given the high prevalence of HIV infection among patients who receive care at the hospital. Although epidemiology of NTM is difficult to define because it is not a reportable disease, the prevalence of NTM has been increasing over the past 30 years [6]–[8]; a high NTM prevalence hampers TB diagnosis by confounding acid-fast bacilli (AFB) smear results.

In 1992, GMH implemented an enhanced respiratory isolation policy that requires airborne infection isolation (AII) of all patients with active TB, TB in the differential diagnosis, and HIV-infected patients presenting with respiratory symptoms and/or an abnormal chest radiograph [9], [10]. During the study period, discontinuation of AII could occur when three AFB smear-negative sputum specimens were obtained and TB was no longer in the differential diagnosis. Since 2002, the GMH Clinical Microbiology Laboratory has routinely performed a NAAT on all AFB smear-positive respiratory specimens. The TB Infection Control Policy at GMH was updated in 2002 to allow patients to be taken out of an AII room if they had an AFB smear-positive respiratory specimen with a negative NAAT without waiting for *Mycobacterium tuberculosis* (MTB) culture results. As part of program evaluation, we conducted a cost analysis study to assess the impact of the routine use of a commercially available NAAT (amplified MTD) on AFB smear-positive respiratory specimens for early exclusion of TB in a "real-world" urban setting where HIV and TB are both prevalent diseases.

## Methods

### Overview/Laboratory Methods

This study was approved by the Emory University Institutional Review Board (IRB) and the Grady Health System Research Oversight Committee. The Emory University IRB approved a request for a waiver of informed consent as the data analyzed for this study were originally collected for infection control and quality improvement purposes. The study took place at GMH and included all patients with an AFB smear-positive respiratory specimen between January 2002 and June 2008. Data on patients with an AFB smear-positive respiratory specimen were collected prospectively. AFB smear and culture were performed on patient respiratory specimens by the GMH Clinical Microbiology Laboratory using standard methodologies; AFB smears were performed using a fluorochrome technique and AFB cultures were performed using both solid and broth-based media [11], [12]. For positive AFB cultures, DNA probes (Accuprobe, Hologic/Gen-Probe) for *M. tuberculosis* complex, *M. avium* complex, *M. kansasii*, and *M. gordonae* were used to identify mycobacterial isolates to the species level. The Amplified MTD (Mycobacterium Tuberculosis Direct) test (Hologic/Gen-Probe) was the NAAT used for *M. tuberculosis*; it was performed according to the manufacturer's recommendations. In the case of indeterminate results, the assay was repeated. For quality assurance the laboratory conducted quality control with positive and negative controls daily. Sensitivity, specificity, positive predictive value (PPV), and negative predictive value (NPV) of the NAAT performed on AFB smear-positive respiratory specimens were calculated using an AFB culture positive for MTB as the reference (gold) standard for diagnosis of TB disease.

### Cost Analysis

**Figure 1:**
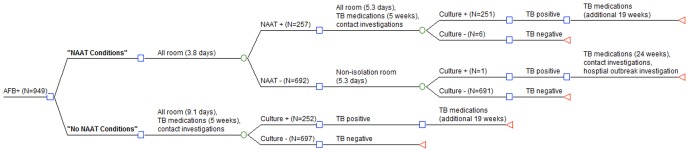
Algorithms for diagnosing tuberculosis with and without a nucleic acid amplification test and associated resources consumed. Definition of abbreviations: AFB = Acid-fast bacillus; AII = airborne infection isolation; NAAT = nucleic acid amplification test; TB = tuberculosis. Legend: The squares represent decision nodes, circles a chance node, and triangles a terminal node. Each condition represents the sequence of events that may occur to patients with an AFB smear-positive respiratory specimen. Using cost inputs described in Table 1, we compared average cost per patient under each condition: “NAAT conditions” versus “no NAAT conditions”.

**Figure 2:**
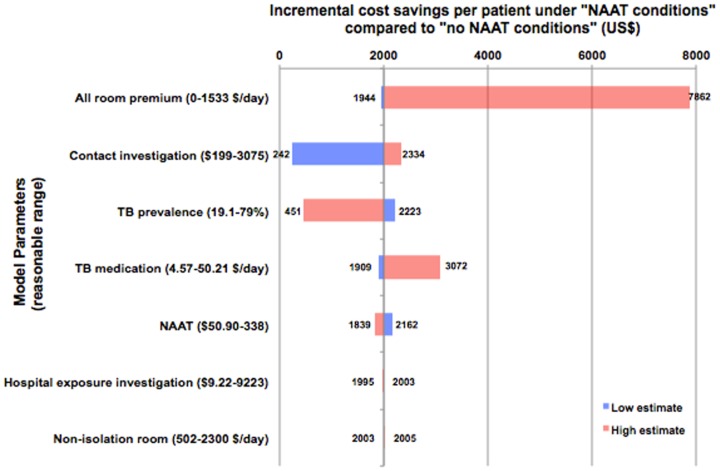
One-way sensitivity analyses with ranges of cost saved due to regular use of a nucleic acid amplification test for TB diagnosis. Definition of abbreviations: AII = airborne infection isolation; NAAT = nucleic acid amplification test; TB = tuberculosis. Legend: Cost ranges for sensitivity analyses are shown next to the given parameter. Numbers next to the bars are estimates for range of cost savings when the given parameter is varied from low (blue bar) to high (red bar) estimates. Costs of smear microscopy and AFB culture are not shown because they are consumed by patients under both “NAAT conditions” and “no NAAT conditions” and therefore do not contribute to cost difference. Base case cost saving with routine use of a NAAT for AFB smear-positive specimens was $2003.

A cost analysis of the routine use of NAAT at GMH was conducted using decision analysis models under two different diagnostic algorithms (**Figure 1**). Decision analysis models were constructed using TreeAge software (TreeAge Software, Inc., Williamstown, MA). Two algorithms were compared: (1) routine use of a NAAT on all AFB-positive smears (“NAAT conditions”), and (2) no use of a NAAT (“no NAAT conditions”). The average cost per AFB smear-positive case under “NAAT conditions” was calculated and compared to hypothetical average cost per AFB smear-positive case under “no NAAT conditions”. One-way sensitivity analyses were conducted to determine sensitivity of cost differences to reasonable ranges of model inputs (**Figure 2**).

A health care system perspective was used; costs that would have been incurred by the entire health care system for patients with an AFB smear-positive respiratory specimen were included. Costs (reported in 2008 U.S. dollars) were assumed to come from laboratory inputs (AFB smear, culture, NAAT), hospital room (either regular room or AII room), anti-TB medications, contact investigations, and hospital exposure investigations (in the case of a false negative NAAT which resulted in healthcare worker exposure to an infectious patient with pulmonary TB who was not in AII precautions) (**Table 1**). Cost estimates were obtained from the GMH Pharmacy and Epidemiology/Infection Control Department, State of Georgia Department of Public Health TB Control Program and Public Health Laboratory, the CDC, and published literature. The cost of the NAAT at GMH was priced at $174 per test based on the cost of the amplified MTD reagent kit ($1,100 for 25 tests including two controls), and technician's labor costs (about 3 hours). The cost of contact investigations was estimated at $2,620 per index TB case (based on costs from a prior study carried out by the Georgia Division of Public Health and CDC in which the cost per contact investigated was $262; in the state of Georgia, each TB case has a mean of 10 contacts). The cost of a hospital exposure investigation was estimated at $92 per episode, based on the cost of a tuberculin skin test at $4.01 and testing a mean of 23 health-care workers (HCWs) exposed to an infectious TB case not properly identified and placed in AII precautions (estimates were obtained from GMH Pharmacy and Epidemiology/Infection Control Department). Due to lack of data on hospital exposure investigation costs from the literature, exposure investigation cost was varied from 1/10 to 100 times the cost of the base case exposure investigation for sensitivity analyses.

**Table 1:** Model parameters, base case and reasonable ranges.

Parameter	Base case	Low	High	Reference (for base case)
**Laboratory costs ($)**				
** Smear microscopy**	3.79	1 [14]	10 [14]	[15]
** AFB culture**	33.10	16.09 [15]	48.96 [15]	[15]
** NAAT**	174	50.90 [3]	338 [2]	Calculation (see text)
**Hospital room cost ($/day)** *				GMH Finance Department
** Non-isolation room**	972.04	502 [14]	2300 [14]	
** AII room premium**	15.35	0 [14]	1533 [14]	
**TB medication ($/day)**	8.23	4.57 [14]	50.21 [15]∧	GMH Pharmacy
**Contact investigation ($)**	2620	199 [18]	3075 [19]	Georgia Division of Public Health (see text)
**Hospital exposure investigation ($)**	92.23	9223†	9.22†	GMH Pharmacy and Epidemiology/Infection Control
**TB prevalence** ‡	26.6%	19.1%#	79% [20]	GMH

AFB = Acid fast bacillus; AII = airborne infection isolation; GMH = Grady Memorial Hospital, Atlanta, GA, USA; NAAT = nucleic acid amplification test; PPV = positive predictive value; TB = tuberculosis

*Base case was determined by multiplying charge by cost/charge ratio.

∧This upper bound was determined in the cited publication by considering all aspects of outpatient care in calculating treatment cost and was included for sensitivity analyses.

† We could not find estimates of exposure investigation cost in the literature, so for sensitivity analyses we took the extreme position of varying the cost from one tenth to 100 times the base case cost (for low and high bounds, respectively).

‡ TB prevalence among patients in our study population, i.e. those with an AFB smear-positive sputum sample. This is equivalent to the PPV of AFB smear microscopy.

#The TB prevalence among AFB smear positives in our study (i.e. PPV of AFB smear) was lower than any found in the literature. For lower bound of TB prevalence we used PPV among HIV patients in our study, i.e. as if all AFB smear-positive patients had HIV.

### Model Building Methods

**(1) “NAAT conditions”:** Under this algorithm (which reflects implementation of the NAAT being performed on all AFB smear-positive respiratory specimens), all patients suspected of having pulmonary TB were placed on AII precautions and admitted to an AII room. Respiratory (sputum) specimens were collected and AFB smears and cultures were performed on respiratory specimens; a NAAT was automatically performed in all cases when a respiratory specimen was AFB smear-positive. The mean turn-around time for a NAAT was 3.8 days as specimens were batched and generally performed twice weekly. Patients were not reported as a TB case to their local health department and a contact investigation was not initiated unless the NAAT was positive.

**(a) NAAT positive:** Those patients with a positive NAAT result were diagnosed with laboratory-confirmed pulmonary TB and kept in an AII room until hospital discharge. These patients were continued on anti-TB medications and were reported as a TB case to their local public health department which then initiated a contact investigation. These patients with pulmonary TB disease were treated for a minimum of 6 months. CDC guidelines state that contact investigations should be initiated no later than 7 business days after reporting of a TB case [13].

**(b) NAAT negative:** Those with an AFB smear-positive respiratory specimen but a negative NAAT were assumed not to have pulmonary TB. After the negative NAAT was reported, AII precautions were discontinued and patients were moved from an AII room to a regular room until hospital discharge. If patients with a negative NAAT were later found to have culture confirmed TB (based on a positive AFB culture for MTB) a hospital TB exposure investigation was conducted.

**(2) “No NAAT conditions”:** Under this hypothetical algorithm (and the situation that existed before implementation of the NAAT in 2002), all patients suspected of having pulmonary TB were admitted to an AII room and had respiratory specimens (sputum) obtained; AFB smears and cultures were performed on respiratory specimens. Those patients with an AFB smear-positive respiratory specimen were kept in an AII room (until hospital discharge), continued on anti-TB medications throughout the hospitalization and at the time of discharge, and were reported as a TB case (or suspect) to their local public health department at the time of the positive AFB smear. The local public health department initiated a contact investigation on all such patients. After discharge, those found to have pulmonary TB on the basis of a positive culture result for *M. tuberculosis* were continued on anti-TB medications for the full course (minimum of 6 months for drug-susceptible TB). Those with a negative culture for *M. tuberculosis* (the culture may have been positive for a NTM) were assumed not to have TB. A contact investigation would have been initiated at the time of the positive AFB smear and carried out before the results of the culture were available based on CDC guidelines [13].

## Results

Among the 1,009 patients with an AFB smear-positive respiratory specimen between January 1, 2002 and June 30, 2008, 949 (94%) had a NAAT performed. Mean age was 44 years, 71% were male, and 84% were African-American. Four hundred forty-five (46.9%) patients were HIV-seropositive, 271 (28.6%) were HIV-seronegative, and 233 (24.5%) had unknown HIV status (i.e., were not tested).

Overall the positive predictive value (PPV) of an AFB smear-positive respiratory specimen for culture-confirmed TB was 27% (252 of 949 respiratory specimens that were AFB smear-positive grew *M. tuberculosis*) (**Table 2**). The PPV of an AFB smear-positive respiratory specimen for culture-confirmed TB was significantly higher for HIV-uninfected persons than for those who were HIV-seropositive (152/271 [56%] vs 85/445 [19%]; RR = 2.94, 95% CI 2.36–3.65, p<0.001). The proportion of patients with a positive AFB smear and negative NAAT increased significantly over time: in 2002 - 55%, 2003 - 62%, 2004 - 68%, 2005 - 81%, 2006 - 83%, 2007 - 85%, 2008 - 73% (p<0.001). Overall the sensitivity of the NAAT for *M. tuberculosis* was 99.6% and the specificity 99.1% (100% sensitivity among HIV-seronegative and 98.8% among HIV-infected patients; 97.5% specificity among HIV-seronegative and 99.2% among HIV-infected patients) (**Table 3**).

**Table 2:** Positive predictive value of an AFB smear-positive respiratory specimen for culture-confirmed tuberculosis stratified by HIV status.

	Positive culture for TB, n	Negative culture for TB (NTM positive), n	Total AFB smear-positive specimens, n	AFB smear PPV, %
**Total**	252	697	949	26.6
**HIV -**	152	119	271	56.1
**HIV +**	85	360	445	19.1
**HIV status unknown**	15	218	233	6.4

AFB = Acid-fast bacillus; HIV+ = HIV-seropositive; HIV - =  HIV-seronegative; NTM = non-tuberculous mycobacteria; PPV = positive predictive value; TB = tuberculosis

**Table 3:** Performance of a nucleic acid amplification test (NAAT) on AFB smear-positive respiratory specimens stratified by HIV status.

		TB culture positive		TB culture negative					
	Patients, N	NAAT +	NAAT −	NAAT +	NAAT −	Sensitivity, %	Specificity, %	PPV, %	NPV, %
**Total**	949	251	1	6	691	99.6	99.1	97.7	99.9
**HIV -**	271	152	0	3	116	100.0	97.5	98.1	100.0
**HIV +**	445	84	1	3	357	98.8	99.2	96.6	99.7
**HIV status unknown**	233	15	0	0	218	100.0	100.0	100.0	100.0

AFB = Acid-fast bacillus; HIV+ = HIV seropositive; HIV- = HIV seronegative; NAAT = nucleic acid amplification test; NPV = negative predictive value; PPV = positive predictive value; TB = tuberculosis; + = positive; − = negative.

### Cost Analysis Results

Patients who had a respiratory specimen that was AFB smear-positive and NAAT-negative were removed from AII precautions and transferred to a regular hospital room. Prior to the availability of the NAAT, all persons who had respiratory specimens that were AFB smear-positive were kept in a AII room while hospitalized, started on empiric anti-TB medications pending culture results, and had their cases reported to the respective local county health department immediately which initiated a TB contact investigation. After the initiation of the NAAT, patients with an AFB smear-positive specimen were reported to their local county health department only if the NAAT was positive.

The mean number of days in an AII room that was averted by a negative NAAT in a patient with an AFB smear-positive respiratory specimen was 5.3 (calculated by the date of discharge minus the day the NAAT test result was available and the patient was transferred from an AII room to a regular room). Additional cost savings were due to avoidance of first line anti-TB medications (isoniazid, rifampin, pyrazinamide, ethambutol, and pyridoxine) among patients that were AFB smear positive, NAAT negative (i.e. because of the NAAT result, there was no need to wait for culture results to “rule out” TB). There were also cost savings for a contact investigation that did not have to be performed on a patient with an AFB smear-positive respiratory specimen that was NAAT negative; the cost of a contact investigation was estimated to be $2,620 per index case (as described in the Methods section).

Inputs for the cost analysis are shown in Figure 1 and Table 1. The average base-case cost per patient with an AFB smear-positive respiratory specimen was $10,219 under NAAT conditions and $12,222 under no NAAT conditions. Testing all AFB smear-positive respiratory specimens with a NAAT resulted in average cost savings of $2003 per patient. One-way sensitivity analyses were robust to ranges of all model parameters; using a NAAT was cost saving compared to not performing a NAAT on an AFB smear-positive respiratory specimen under reasonable ranges of all parameters (**Figure 2**). Routine use of the NAAT on AFB smear-positive respiratory specimens was highly cost saving.

## Discussion

Our study is one of the first to demonstrate cost savings by routine use of a commercially available and FDA-approved NAAT (performed on an AFB smear-positive respiratory specimen from every patient who had a sputum/respiratory specimen that was AFB smear-positive) in a hospital setting in the U.S. The NAAT is recommended for routine use on respiratory specimens by the CDC [1]. Several previous studies of the costs of commercial NAAT for use in high-income countries (Finland, UK and US) concluded that routine use of NAAT was not cost effective in low TB prevalence settings, but sensitivity analyses in those studies suggested that it might be cost-effective at higher prevalence rates of TB in the target population [2]–[4]. Two recent studies using hypothetical cohorts reported that a recently FDA-approved NAAT, Xpert MTB/RIF, could be cost effective in the U.S.; in one study this was due to improved AII room usage [14], [15]. In our study, we explored the cost impact to the health care system of performing the NAAT and found the test to demonstrate substantial cost savings, in large part due to avoidance of performing contact investigations among patients who were AFB smear-positive but did not have TB. Prior to the implementation of the NAAT, all patients who had a AFB smear-positive respiratory specimen were reported to their local health department and considered to have TB, which initiated a contact investigation. Only several weeks later after culture results were back, could it be determined whether the patient had TB or the culture was positive for a NTM.

We found a low PPV of an AFB smear for culture-confirmed TB at our institution (27%), especially among HIV-seropositive patients (<20%), which was due to the high prevalence of positive respiratory cultures for NTM among HIV-infected persons. TB infection control policies at our hospital are focused on detecting all TB cases and not missing any cases of TB among patients, including those with HIV infection. The low PPV of the AFB smear for TB was a major factor in the cost savings of the NAAT, although sensitivity analyses demonstrated cost savings even with a higher PPV. In addition, the utility of the NAAT improved the use of AII rooms by allowing patients admitted to AII rooms who had respiratory specimens that were AFB smear positive but did not have TB to be moved into regular rooms more rapidly. While the TB infection control program at GMH has been highly efficacious in protecting patient and health care worker safety by preventing nosocomial transmission of TB, it results in over-isolation of patients in AII rooms admitted with pulmonary symptoms and/or abnormal chest radiographs [9], [10], [16], [17]. The isolation of patients in AII rooms drives the number of sputum specimens collected. The NAAT allowed for more efficient use of AII rooms.

The NAAT performed extremely well among both HIV-infected and HIV-seronegative patients with extremely high sensitivity and specificity. Early exclusion of TB (especially among those who were HIV-seropositive) allowed early discharge from AII rooms, avoidance of unnecessary anti-TB therapy, and avoidance of the initiation of contact investigations by local public health departments among those who were AFB smear-positive but turned out not to have TB (i.e., NAAT negative respiratory specimen and subsequently confirmed to be AFB culture negative). Routine use of NAAT on AFB smear-positive respiratory specimens was shown to be cost-savings in our setting (>$2000 per test performed).

Our study is subject to several limitations. At our hospital, the NAAT is routinely performed only on AFB smear-positive respiratory specimens so we were not able to assess the cost effectiveness of the NAAT when performing the test on all specimens (i.e. including AFB smear-negative specimens). Performing the test on all respiratory specimens could potentially have led to different results in our cost analysis. In addition, our hospital cares for large numbers of HIV-infected patients and more cases of TB than any other hospital in Atlanta or the state of Georgia, so care must be employed in generalizing our findings to other settings. Because we used our patients as their own control (“no NAAT conditions”), we were not able to assess the impact of the NAAT on reducing hospital stay and therefore may have underestimated cost savings.

In conclusion, we found the NAAT to be highly sensitive and specific (>99% for each) on AFB smear-positive specimens compared to culture. The overall PPV of an AFB smear-positive respiratory specimen for culture-confirmed TB was low at our hospital (27%), in large part because of a low PPV among HIV-infected patients. Routine use of the NAAT on AFB smear-positive respiratory specimens was highly cost-saving (saved >$2000 per AFB smear-positive case) in our setting at a U.S. urban public hospital with a high prevalence of TB and HIV.
